# Development of a hypoallergenic and immunogenic Pru p 3 proline variant for treatment of peach allergy

**DOI:** 10.1186/2045-7022-4-S2-O22

**Published:** 2014-03-17

**Authors:** Stephanie Eichhorn, Markus Steiner, Josef Laimer, Peter Lackner, Laurian Zuidmeer-Jongejan, Peter Briza, Adriano Mari, Ronald van Ree, Fatima Ferreira, Gabriele Gadermaier

**Affiliations:** 1 University of Salzburg, Department of Molecular Biology, Division of Allergy and Immunology, Salzburg, Austria; 2 Academic Medical Center, Department of Experimental Immunology, Amsterdam, Netherlands; 3 Associated Centers for Molecular Allergology, Associated Centers for Molecular Allergology, Rome, Italy

## Background

Allergic reactions to peach are highly prevalent in the Mediterranean area with persistent and potentially life threatening symptoms. We aimed to develop a save and efficient immunotherapeutics for treatment of peach allergy by targeting the major allergen Pru p 3.

## Methods

We deployed an *in-silico* mutagenesis approach to design a fold variant of recombinant Pru p 3. Four stability hot spot residues were identified and proline was predicted as the most effective replacement amino acid. Pru p 3 C1 was produced in *E.coli* using a pET-based expression system. After refolding and purification by cation exchange chromatography, the protein was characterized by reducing and non-reducing gel electrophoresis, circular dichroism spectroscopy, size exclusion chromatography, dynamic light scattering and mass spectrometry (MS). An accelerated stability test was performed by storing the protein up to 6 month at temperatures ranging from -70°C to +40°C.The IgE binding capacity of Pru p 3 C1 and WT Pru p 3 was tested in ELISA using sera from peach allergic patients. Different adjuvants and adsorption conditions were evaluated for use in a mouse model. Mice were s.c. immunized with Pru p 3 C1 and WT Pru p 3 and sera were analyzed for IgG reactivity.

## Results

Recombinant Pru p 3 C1 was produced with a yield of 15 mg/l expression volume. The purity and identity of the protein was confirmed in gel electrophoresis and MS. In size exclusion chromatography and dynamic light scattering the protein showed to be >90% monomeric. Circular dichroism spectroscopy showed that the protein is predominantly in an unfolded state. Thermal denaturation led to a shift of the spectrum which reverted to the initial curve after renaturation. After six month of storage Pru p 3 C1 demonstrated high stability and no aggregation or degradation behavior at temperatures ranging from -70°C to +4°C. In ELISA, the IgE binding capacity of Pru p 3 C1 was reduced by 89% compared to WT Pru p 3. *In vivo* models showed that the use of aluminum phosphate as adjuvant is superior over aluminum hydroxide. Immunization with Pru p 3 C1 induced a humoral immune response in all animals and triggered cross-reactive IgG1 antibodies in 50% of mouse sera.

## Conclusion

Pru p 3 C1 shows high stability, strongly reduced IgE binding capacity and immunogenicity. Therefore, the molecule is a very promising candidate for treatment of peach allergy.

The study was supported by the FAST project EU grant 201871.

